# High Risk Sexual Behaviors for HIV among the In-School Youth in Swaziland: A Structural Equation Modeling Approach

**DOI:** 10.1371/journal.pone.0067289

**Published:** 2013-07-04

**Authors:** Hlengiwe Nokuthula Sacolo, Min-Huey Chung, Hsin Chu, Yuan-Mei Liao, Chiung-Hua Chen, Keng-Liang Ou, Lu-I Chang, Kuei-Ru Chou

**Affiliations:** 1 Graduate Institute of Nursing, College of Nursing, Taipei Medical University, Taipei, Taiwan; 2 Institute of Aerospace and Undersea Medicine, School of Medicine, National Defense Medical Center, Taipei, Taiwan; 3 Department of Neurology, Tri-Service General Hospital, National Defense Medical Center, Taipei, Taiwan; 4 School of Nursing, Mei-Ho University, Pingtung, Taiwan; 5 Graduat Institute of Biomedical Materials and Tissue Engineering, College of Oral Medicine, Taipei Medical University, Taipei, Taiwan; 6 Research Center for Biomedical Devices and Prototyping Production, Taipei Medical University, Taipei, Taiwan; 7 Research Center for Biomedical Implants and Microsurgery Devices, Taipei Medical University, Taipei, Taiwan; 8 Department of Dentistry, Taipei Medical University-Shuang-Ho Hospital, Taipei, Taiwan; Research and Development Corporation, United States of America

## Abstract

**Background:**

Global efforts in response to the increased prevalence of the human immunodeficiency virus (HIV) are mainly aimed at reducing high risk sexual behaviors among young people. However, knowledge regarding intentions of young people to engage in protective sexual behaviors is still lacking in many countries around the world, especially in Sub-Saharan Africa where prevalence of human immunodeficiency virus is the highest. The objective of this study was to test the theory of planned behavior (TPB) for predicting factors associated with protective sexual behaviors, including sexual abstinence and condom use, among in-school youths aged between 15 and 19 years in Swaziland.

**Methods:**

This cross-sectional survey was conducted using a anonymous questionnaire. A two-stage stratified and cluster random sampling method was used. Approximately one hundred pupils from each of four schools agreed to participate in the study, providing a total sample size of 403 pupils of which 369 were ultimately included for data analysis. The response rate was 98%. Structural equation modeling was used to analyse hypothesized paths.

**Results:**

The TPB model used in this study was effective in predicting protective sexual behavior among Swazi in-school youths, as shown by model fit indices. All hypothesized constructs significantly predicted intentions for abstinence and condom use, except perceived abstinence controls. Subjective norms were the strongest predictors of intention for premarital sexual abstinence; however, perceived controls for condom use were the strongest predictors of intention for condom use.

**Conclusions:**

Our findings support application of the model in predicting determinants of condom use and abstinence intentions among Swazi in-school youths.

## Introduction

Human immunodeficiency virus (HIV) infection is a global crisis that represents a serious health threat, particularly among younger people. Worldwide, almost six thousand young people (aged between 15 and 24 years) contract HIV on a daily basis, and over a quarter of all individuals living with HIV are aged between 15 and 24 years [1]. Most countries with generalized epidemics report that major declines in HIV infection rates are associated with changes in adolescent sexual behaviors, mainly including delayed sexual debut, consistent condom use and reduced number of sexual partners [2]. It is concerning, therefore, that young people in most countries continue to engage in high risk sexual behaviors despite the presence of robust HIV prevention strategies aimed at reducing such behaviors [3]. Among young people, the consequences of engaging in sexual risk taking behavior have been well documented and include sexually-transmitted infections (STIs), abortion, pregnancy and increased likelihood of dropping out from school [4], [5].

Socio-cognitive studies have been successful in helping to explain why individuals engage in certain behaviors. Indeed, the theory of planned behavior (TPB) has been one of the most commonly used techniques in studies that have focused on younger people [6]–[8]. The TPB theory promotes health preserving behaviors by addressing structural and individual factors that underlie the intention to engage in high risk sexual behavior. Apart from the known influence of individual attitude and perceived behavioral control [9]–[11], this theory allows the influence of subjective norms on health related behaviors to be studied.

Subjective norms such as culture, parental influences, and peer norms are emerging as major predictors of sexual behavior. Behavioral data provide evidence that factors such as societal organizational rules, an individual’s societal role, an individual’s extent of power/influence in society and hierarchical level, all exert remarkable effects on the behavior of the individual [12]–[14]. However, such factors appear to have varying levels of influence that are country specific; in most African societies, evidence indicates that adolescents prefer and respond the most strongly to the opinions of their community leaders and their families [15]. African societies uphold premarital abstinence as the ideal form of protective sexual behavior among young people; however, modernization may be reducing the power of this norm to promote sexual health, leading to increased HIV infection rates. In Swaziland, for example, the concept of marriage no longer holds the same value as in previous generations, which can be explained in part by the devastating effects of HIV on family structures and the subsequent increase in single parent families or orphaned children being cared for by grandparents or older siblings [16]. Young people may therefore be less motivated to abstain from premarital sex with a view to waiting until marriage. In addition, young people in such societies tend to hide their sexual relationships in order to avoid society’s harsh judgments, exacerbating their participation in riskier sexual practices [17].

Failure of young people to abstain from sex has led to condom use becoming a major focal point in many studies of human sexual behavior [18]. The increasing recognition of the need for alternate strategies, however, presents serious challenges in extremely conservative societies that deny the reality of sexual activity among young people. Indeed, parents within such societies often find it difficult to talk to their children about contraception, despite evidence that they can have a remarkably positive influence on adolescents’ sexual behavior [19]–[21].

Existing literature has established peer pressure as another powerful influence on adolescent sexual behavior [22], [23], although this tends to be negative, being more likely to perpetuate engagement in high risk sexual practices. Survey data revealed that sexual information sources such as friends, cousins and the media were associated with increased adolescent intentions to initiate sexual intercourse. In contrast, the opposite was found for information sources such as parents, grandparents and religious leaders [24]. Moreover, the susceptibility of adolescents to peer influence tends to vary by the strength of parent-child attachment, such that adolescents who have stronger social bonds with their parents may be less prone to peers influence [25]. The lack of contextual findings specific to African countries may therefore result in youth programs that over-rate the influence of peers on adolescent sexual behavior, while underestimating the effect of parental and community norms on the promotion of safer sexual practices. The purpose of this study was therefore to examine contextual factors underlying protective sexual behaviors, focusing specifically on (1) abstinence and (2) condom use among Swazi in-school youths aged between 15 and 19 years.

## Materials and Methods

This was a cross-sectional survey utilizing anonymous and self-reported questionnaires to predict protective sexual behaviors among Swazi in-school youths.

### Participants and Sampling Procedures

The study focused on high school youths aged between 15 and 19 years, who were at junior and senior levels in public high schools located in the northern region of Swaziland. Teachers assisted in the recruitment process during daily morning assemblies, which was undertaken two weeks before data collection. Day pupils were eligible for participation if they were Swazi, aged between 15 and 19 years, and enrolled at high school level. Participation was voluntary and parental consent was necessary for individuals younger than 18 years. Excluded cases included those not within the stated age range, those without parental consent, non-Swazis and pupils residing at boarding schools. Three hundred and sixty nine of 403 participants were included in the data analysis. After excluding missing data (3.0%), participants without parental consent (1.7%), and those over the age of 19 years (3.7%), 369 individuals were included in our final analysis. Justification for the sample size was based on the rule of thumb in structural equation modeling (SEM) that asserts that the sample should include at least 10 to 15 cases per measured indicator [26]. In the current study, the structural equation model measured 15 variable indicators; thus, the sample size of 369 pupils was appropriate to test the model.

This study employed a two-stage stratified random sampling method. The first stage was stratified random sampling, which involved classification of schools according to rural and urban strata. At the time of this study, there were 204 public high schools in Swaziland, defined as schools that include Forms 4 and 5 or Grades 9 and 10 [27]. Classification of the schools according to the four regions of Swaziland was as follows; Hhohho  = 55, Manzini = 55, Lubombo = 45, Shiselweni = 49. The Hhohho region of Swaziland was chosen as the study’s area of interest since it not only has a higher number of schools but has also been experiencing a higher prevalence of HIV despite recent declines (Hhohho = 21%, Manzini = 19%, Shiselweni = 16%, Lubombo = 19%) [28], [29]. The list of high schools in this region served as the sampling frame [27]. Schools were stratified according to their location in rural or urban areas; 38 were rural and 17 were urban [27]. Four schools were randomly selected from those in urban areas and the same for those in rural areas. The second stage was cluster random sampling, whereby two classes were chosen at random from the list of all classes that taught Forms 4 and 5.

### Measures

A total of 8 different instruments were used in this study. The first instrument measured variables relating to background and sexual behavior of participants [30] which had 34 items related to eligibility screening, demographic information and history of sexual behavior. Since this questionnaire was originally developed for Korean college students, it was modified in this study in order to suit Swazi in-school youth. Questions relating to employment, income and military service were excluded and three items were added relating to class level, name of school and the region in which the student lives. The latter 7 instruments were derived from variables of the Theory of Planned Behavior and relate to premarital sex and condom use. These instruments were originally developed by different authors, however; those used in this study were modified Cha and his associates [30], [31].

#### Premarital Sexual Attitude Scale

Attitudes toward premarital sex were measured by a modified Premarital Sexual Attitude (PSA) Scale which consisted of 20 items measured on a 4-point Likert scale. Adolescent acceptance of sexual behavior (e.g. kissing, light and heavy petting, sexual intercourse), ranging from “strongly agree” (1) to “strongly disagree” (4), was assessed at five relationship levels (sexual worker, casual partner, steady partner, lover, and fiancée). High scores indicated favorable attitudes toward abstinence. Cronbach’s alphas for the modified and original scales were 0.94 and 0.89, respectively [7], [31]. In this study, Cronbach’s alpha was 0.93 among Swazi high school students.

#### Condom Use Attitude Scale

A modified Condom Attitude Scale (CAS) was used to measure attitudes toward condom use. This scale had 16 items measured on a 5-point scale ranging from “strongly disagree” (1) to “strongly agree” (5) [32]. Higher scores implied positive attitudes toward condom use. Cronbach’s alpha in the current study was 0.90, comparable with the value given in Cha’s study (also 0.90) among Korean college students [32].

#### Referent Group Norms of Sexual Behavior Scale

Premarital sex subjective norms were measured by a modified referent group norm of Sexual Behavior Scale [7], which consisted of 20 items measured on a 5-point Likert scale ranging from “strongly approve” (1) to “strongly disapprove” (5). Premarital sex subjective norms (kissing, light and heavy petting, sexual intercourse) were assessed in relation to four sub-domains (mother, father, culture, and friend norms) at five relationship levels (sex worker, casual partner, steady partner, lover, and fiancée). Higher scores indicated greater social pressure toward premarital abstinence. In Cha’s study, Cronbach’s alphas ranged from 0.84 to 0.90 [7]. In the current study, Cronbach’s alphas for the four sub-domains were higher, ranging from 0.94 to 0.98.

#### Referent Group Norms of Condom Use Scale

The modified referent group norms of Condom Use Scale [32] was used to assess subjective norms of condom use. It consisted of 12 items measured on a 5-point Likert scale ranging from “definitely no” (1) to “definitely yes” (5). This scale assessed four types of norms including mother, father, partner, and friend norms, each consisting of three items (partner norms, however, were excluded from analysis because not all participants had sexual partners). The scale showed good reliability among US adolescents and Korean students, with reliability scores of 0.84 and 0.74, respectively [32], [33]. In the current study, Cronbach’s alphas for the three sub-domains of mother, father, and friend norms ranged from 0.70 to 0.87. Higher scores indicated higher approval for condom use.

#### Sexual Abstinence Self-efficacy Scale

To examine perceived behavioral control of abstinence, the Norris Sexual Abstinence Self-efficacy (ASE) Scale was used. This scale consisted of 7 items. Cronbach’s alpha was 0.83 in Norris’s study [34] and 0.85 in the current study. The instrument’s response options ranged from “not at all sure” (1) to “extremely sure” (4). Higher scores revealed higher levels of motivation to abstain from premarital sex.

#### Condom Self-efficacy Scale

A modified Condom Self-efficacy Scale was used to measure perceived behavioral control of condom use [32]. This scale had three sub-domains (consistency, correct use, and communication). Cha and Hanna measured the reliability of the scale among US adolescents and Korean college students, obtaining scores of 0.85 and 0.91, respectively [32], [35]. The response options for this scale ranged from “very unsure” (0) to “very sure” (4). Higher scores indicated greater perceived self-control over condom use. The reliability of the scale in the current study was 0.87 for the total 15 items, while reliability scores for the sub-domains ranged from 0.78 to 0.81.

#### Intention of Sexual Behavior Scale

Intention was measured using Doswell’s Modified Intention of Sexual Behavior Scale [7] [32]. Participants were asked whether they intend to engage in premarital sex and whether they intend to use condoms whenever they engage in premarital sex. Cha’s version consisted of 25 items measured on a 4-point Likert scale ranging from “strongly disagree” (1) to “strongly agree” (4). The first 20 items measured intentions for premarital abstinence (IPS) and the final 5 items measured intentions for condom use (IPC). The Cronbach’s alphas for the modified and original scales were 0.96 and 0.84, respectively [7], [32], [36]. Higher scores revealed greater intentions to abstain from sexual intercourse and greater intentions for condom use in future sexual encounters.

### Ethics Statement

The study protocol was approved by the Taipei Medical University Joint Institutional Review Board. Permission to carry out the project was also sought from the Swaziland Scientific and Ethics Committee (SEC), Ministry of Education, the Regional Education Officer, and principals in the selected schools. All participants gave their informed consents in writing, together with written permissions from parents of pupils aged less than 18 years. The composite questionnaire was administered by project staff directly to participants in a classroom setting. Students were allowed up to one hour in class to complete the anonymous questionnaire under exam conditions. After completion, questionnaires were placed and sealed in an envelope provided by project staff. Refusal to complete the questionnaire, even after informed consent had been given, was allowed. Class teachers were not present during completion of the questionnaires.

### Data Analysis

Descriptive statistics were performed on socio-demographic variables and all instruments based on TPB constructs (premarital attitude, condom attitude, norms of sexual behavior, norms of condom use, abstinence self-efficacy, condom self-efficacy, intention to use condoms, and intention to abstain from premarital sex) using SPSS version 19.0 for Windows (SPSS, Inc, Chicago, IL). SEM was performed with AMOS 20.0 to analyze all hypothesized paths of the model. A number of individual items in PSA, ASE, IPS, CAS, and IPC were summed up to construct item parcels as indicators of the latent variables. The parceled data were less likely to have correlated residuals or multiple cross-loadings [37]. Model fit analysis was performed using the following fit indices and their cut-off points: χ^2^/df = 2.00; goodness of fit index (GFI) = 0.90; comparative fit index (CFI) = 0.90, and root mean squared error of approximation (RMSEA) = 0.05 [38].

## Results

### Descriptions of the Sample and Sexual Behaviors

The proportions of participants were almost equivalent in terms of gender (53.1% male and 46.9% female), area of residence (49.6% urban and 50.4% rural) and Grade level (55.8% junior high school and 44.2% senior high school). The sample was predominantly Christian (98.1%). Over three quarters of the students had at least one boyfriend or girlfriend (77.8%), almost a quarter of the sample were non-virgins (41.5%), and 65% of individuals had the perception that their friends engaged in sexual intercourse. The mean age of first sexual intercourse was 15.90±1.43 years, ranging from 12 to 19 years. Among students who were non-virgins, almost three-quarters (74.6%) had not used condoms at first sexual intercourse.

### Description of Study Variables

Mean values, ranges and standard deviations (SD) were calculated for all observed variables (Table1 and Table 2). Among premarital sex norms, mother and father norms (standardized item mean = 4.09, mean = 4.01) were weighted most heavily toward abstinence, followed by culture norms (mean = 3.62), and peer norms (standardized item mean = 2.65). In contrast, regarding condom use subjective norms, the opposite was found. Peer norms of condom use were more heavily weighted toward the use of condoms (standardized item mean = 3.77) compared with mother (standardized item mean = 3.14) and father (standardized item mean = 2.79) norms. There was high variability of responses for father norms of abstinence (SD = 0.96). In summary, all TPB variables scored above average and showed minimal to high variability of responses.

**Table 2:** Descriptive statistics of observed variables.

Questionnaire	Total items	Range	S-IM	SD
Premarital attitude scale	20	20–80	2.62	0.56
Condom attitude scale	16	27–80	3.65	0.79
Subjective norms of sexual abstinence:				
Father norms	20	32–100	4.01	0.96
Mother norms	20	32–100	4.09	0.87
Friend norms	20	20–100	2.65	0.78
Culture norms	20	32–100	3.62	0.79
Subjective norms of condom use:				
Father norms	3	3–15	2.79	0.81
Mother norms	3	3–15	3.14	0.77
Friend norms	3	3–15	3.77	0.90
Abstinence self efficacy	7	7–28	2.87	0.77
Condom self efficacy	15	11–60	2.48	0.80
Intention to abstain	20	20–80	2.81	0.61
Intention for condom use	5	5–20	2.54	0.78

Abbreviation: S-IM = standardized item mean.

**Table 1:** Descriptive data of study participants (n = 369).

Variable	n	%
**Sex**		
Male	196	53.1
Female	173	46.9
**Area of residence**		
Urban	183	49.6
Rural	186	50.4
**Religion**		
Christian	362	98.1
Muslim	2	0.5
Other	5	1.4
**Grade level**		
Form 4	206	55.8
Form 5	163	44.2
**Current boy/girlfriend**		
Yes	287	77.8
No	82	22.2
**Sexual experience**		
Virgin	215	58.5
Not virgin	153	41.5
**Friends engaged in sex**		
Most of them	100	27.1
Half of them	41	11.1
A few of them	99	26.8
None of them	129	35.0
**Protection at first sex (n = 153)**		
None	98	64.1
Coitus interruptus	15	9.8
Oral pills	1	0.7
Condom	39	25.5

### The Original Model of TPB Protective Sexual Behaviors

**Figure 1:**
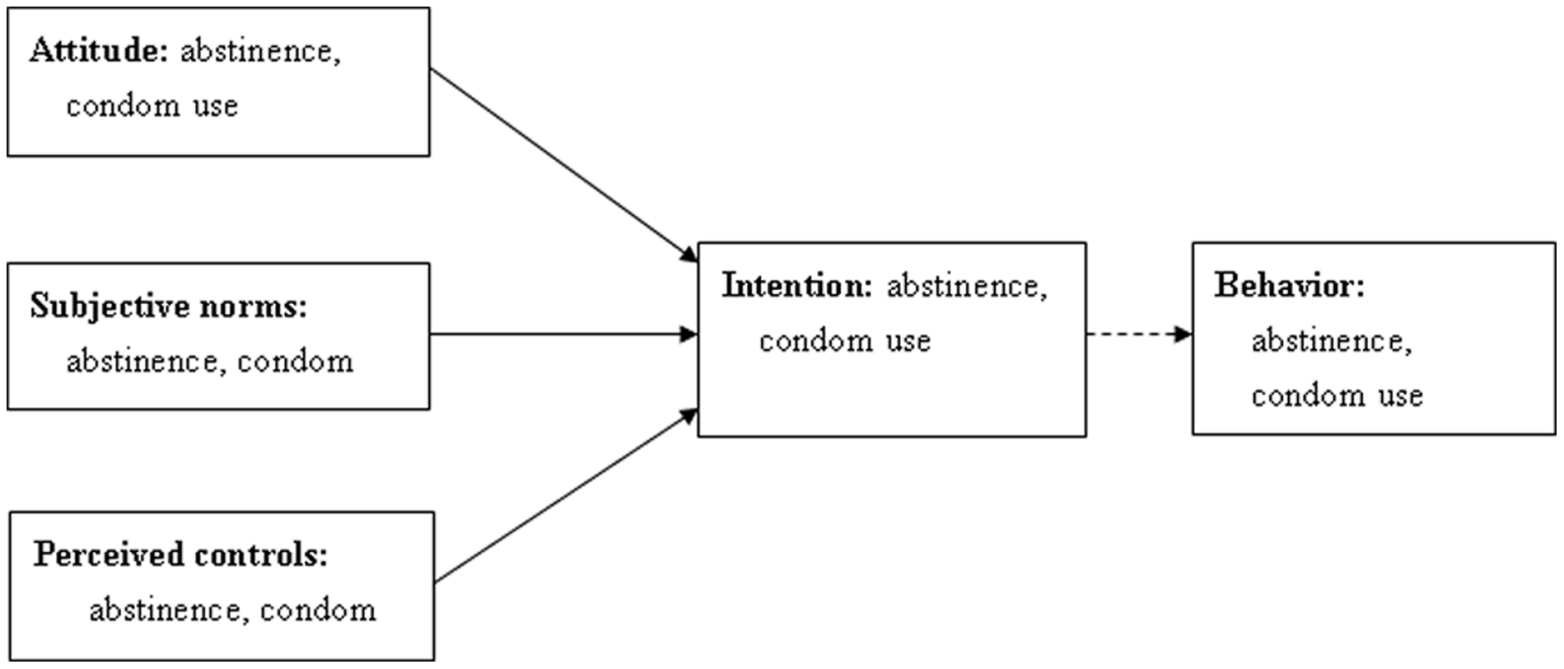
Schematic representation of measured variables in the current study: exogenous and endogenous variables.

**Figure 2:**
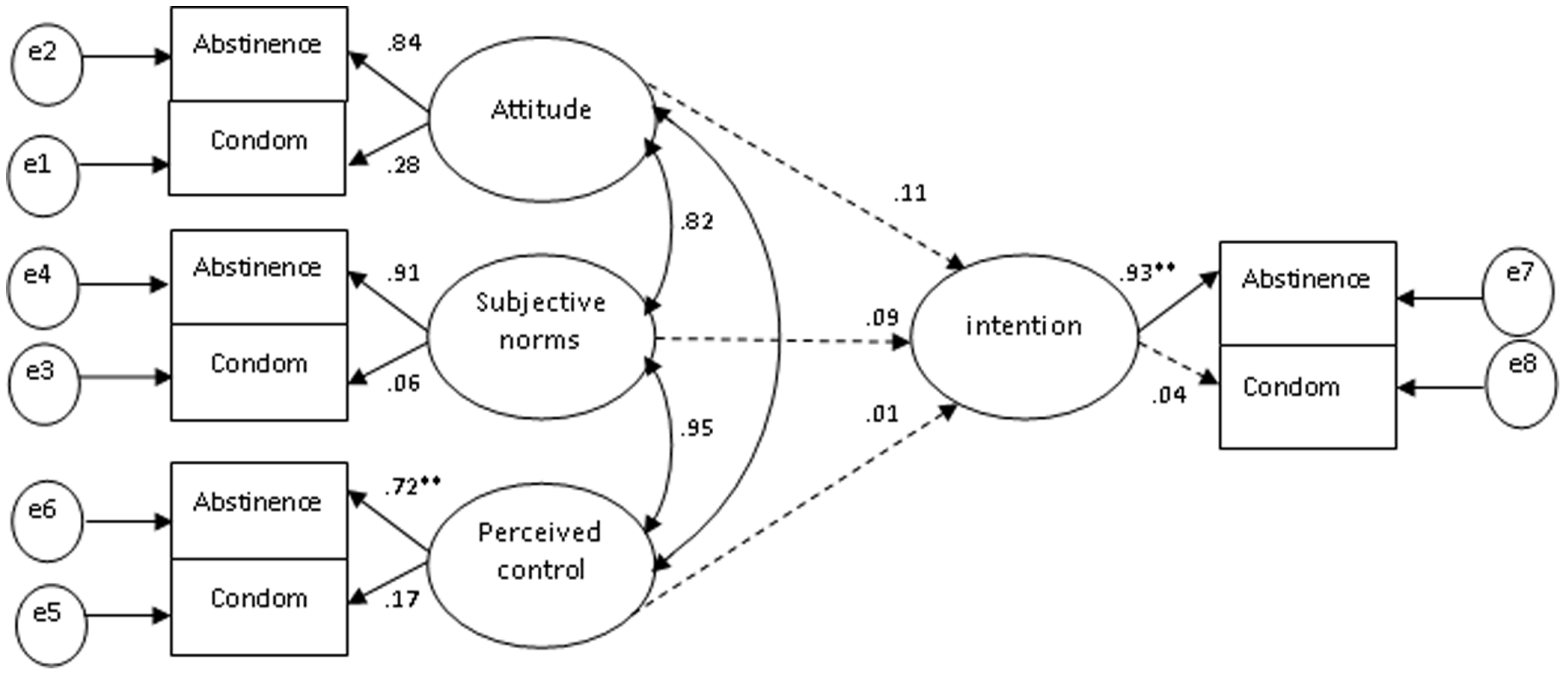
Original model of TPB protective sexual behaviors. Model fit indices: χ^2^/df = 33.47, GFI = 0.72, CFI = 0.66, RMSEA = 0.30, broken line = non-significant path. **p<0.01.

A hypothetical model was examined in the full sample using SEM. First, the TPB was translated into a basic statistical model (Figure 1 and Figure 2). This model did not fit with the data, as indicated by poor fit indices (χ^2^/df = 33.47, GFI = 0.72, CFI = 0.66, and RMSEA = 0.30) and non-significant paths. The reason for this poor fit could be due to use of only two indicators for measuring each of the constructs of the theory of planned behavior (attitude, subjective norm, and perceived behavioral control) and the minimal or absent correlations among such indicators. According to Kline [39], indicators measuring the same latent variable should at least be moderately correlated, especially in cases where the number of indicators per latent variable is less than three.

### The Final Model of TPB Protective Sexual Behaviors

**Figure 3:**
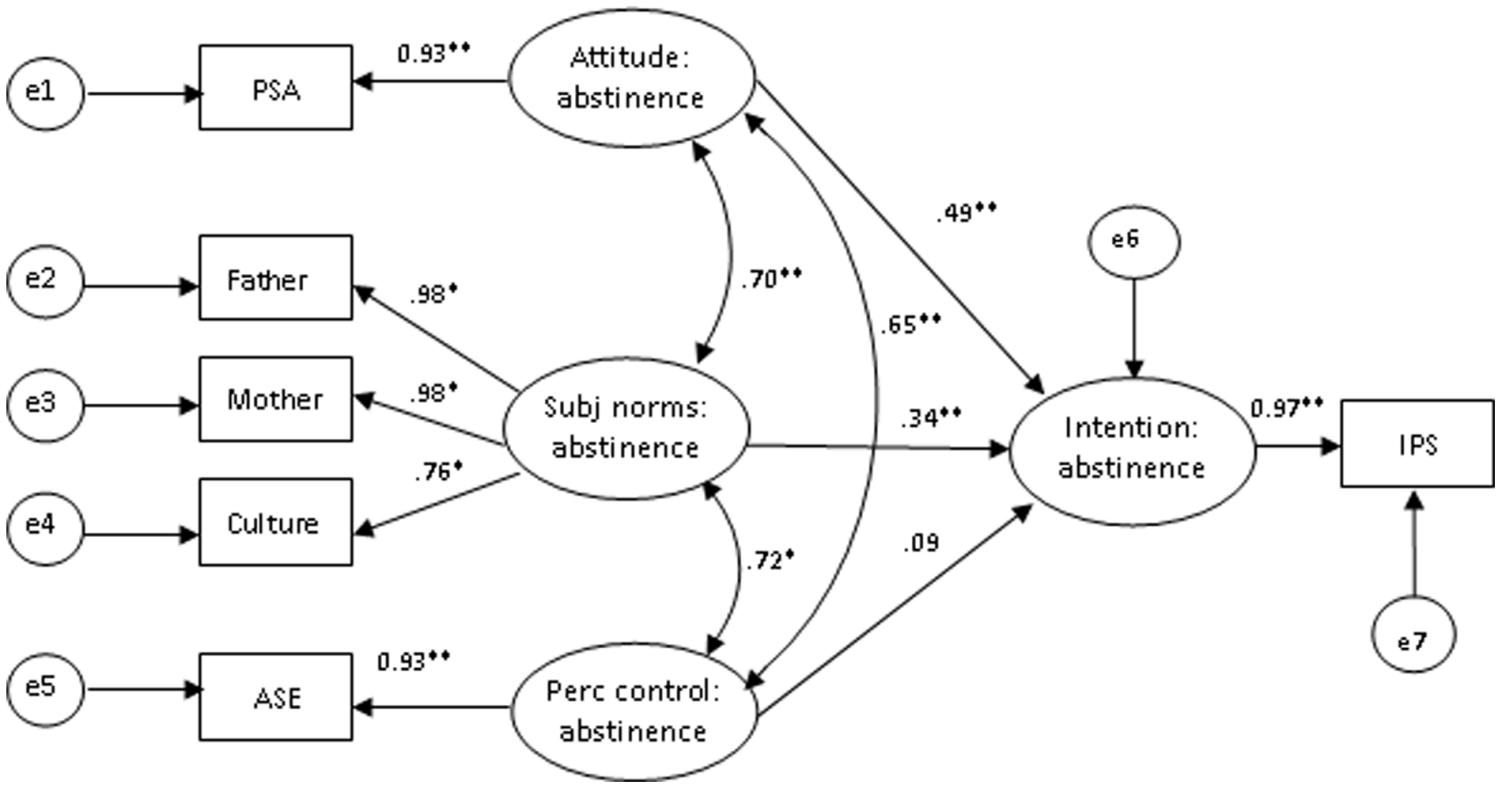
The model of TPB intention for premarital sexual abstinence. Model fit indices: χ^2^/df = 2.257, GFI = 0.988, AGFI = 0.958, CFI = 0.996, NFI = 0.993, RMSEA = 0.058. Abbreviations: PSA = premarital sexual attitude scale, ASE = abstinence self efficacy, IPS = intention for premarital sexual abstinence. *p<0.05, **p<0.01.

**Figure 4:**
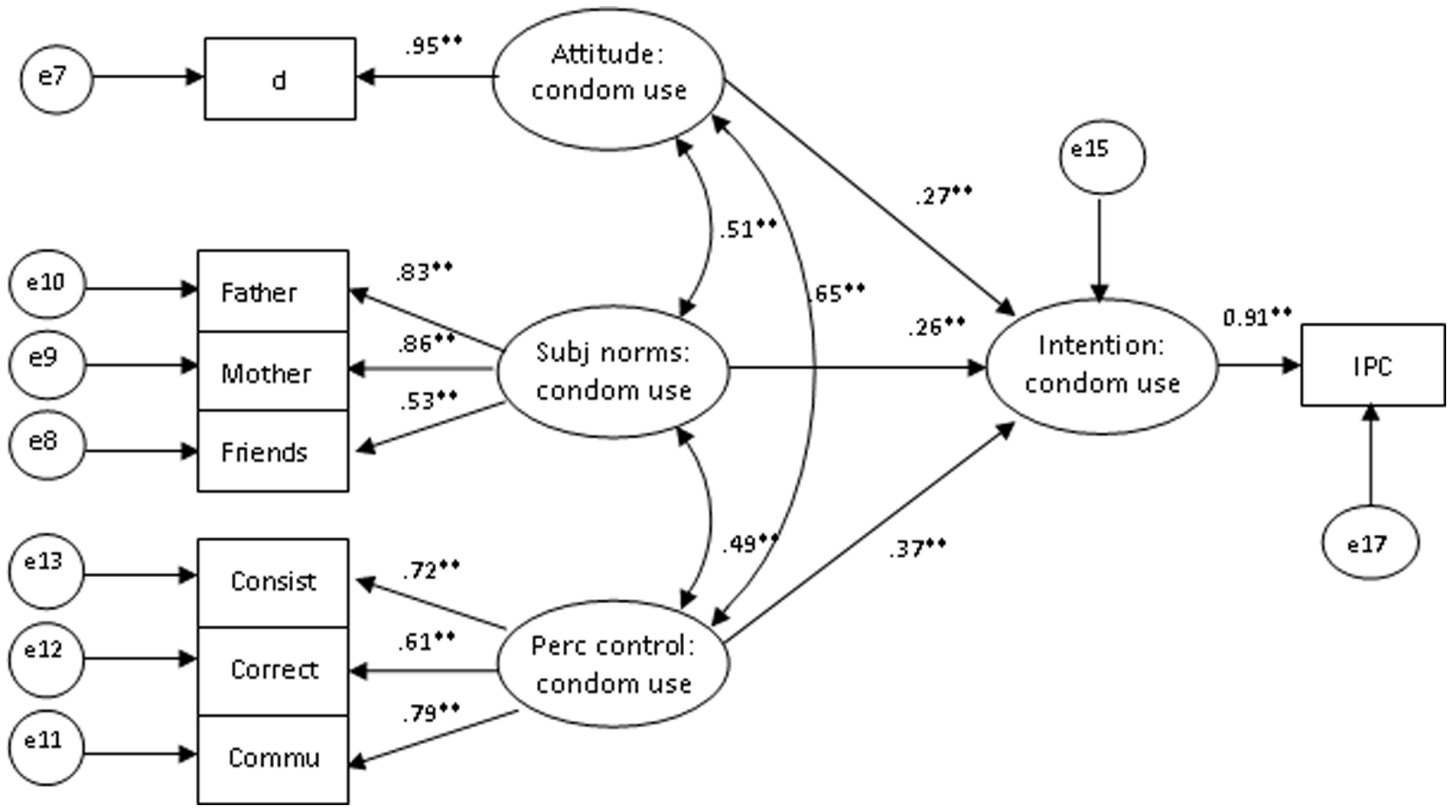
The model of TPB intention for condom use. Model fit indices: χ^2^/df = 3.034, GFI = 0.970, AGFI = 0.933, CFI = 0.970, NFI = 0.956, RMSEA = 0.074. Abbreviations: CAS = condom attitude scale, IPC = intention for premarital condom use, consist = consistency in condom use, correct = correct use of condoms, commu = communication on condom use. **p<0.01.

The final model culminated from re-specification of the model by splitting the non-correlated indicators that measure protective sexual intentions. This led to drastic improvement in hypothesized paths and model fit indices (χ^2^/df = 2.257, *p = *0.035, GFI = 0.988, AGFI = 0.958, CFI = 0.996, NFI = 0.993, RMSEA = 0.058) in the model of intentions for premarital sexual abstinence (Figure 3). The model fit indices (χ^2^/df = 3.034, *p*<0.01, GFI = 0.95, AGFI = 0.95, CFI = 0.97, NFI = 0.956, RMSEA = 0.074) were shown in the model for intentions to use condoms (Figure 4). This model then became the final model since all paths contained within it were statistically significant and generally consistent with theoretical expectations.

Both abstinence and condom use intentions were significantly predicted by all hypothesized constructs of the TPB. The strengths of the predicted relationships in the model for premarital sexual abstinence and condom use were 63.7% and 43.8%, respectively. Subjective norms were the strongest predictors of intention to abstain (effect coefficient = 0.41, *p*<0.01), of which the most influential were mother and father norms followed by culture norms. Favorable premarital sex attitudes also promoted sexual abstinence (effect coefficient = 0.39, *p*<0.01), although perceived abstinence controls were the weakest predictors of abstinence intentions (effect coefficient = 0.10, *p = *0.146). Regarding condom use intentions, perceived controls were the strongest predictors (effect coefficient = 0.36, *p*<0.01), followed by subjective norms of condom use (effect coefficient = 0.22, *p*<0.01) and attitudes (effect coefficient = 0.22, *p*<0.01). (Table 3).

**Table 3:** Path coefficient for final model of planned behaviors.

Path	USC	SC	SE	t-value
**Structural model:**				
**TPB intention for premarital sexual abstinence**				
Attitude-abstinence and intention-abstinence	0.542	0.493	0.063	8.563*
Subj. norms of abstinence and intention-abstinence	0.020	0.343	0.003	6.028*
Perc. control-abstinence and intention-abstinence	0.093	0.092	0.060	1.549
**TPB intention for condom use**				
Attitude-condom use and intention-condom use	0.233	0.270	0.061	3.835*
Subj. norms of condom use and intention-condom use	0.083	0.261	0.019	4.306*
Perc. control-condom and intention-condom use	0.116	0.371	0.025	4.708*

Abbreviations: subj. norms = subjective norms, perc. control = perceived control, USC = un-standardized path coefficient, SC = standardized solution, SE = standard error. *p<0.01.

## Discussion

### Major Socio-demographic Findings

In Swaziland, the prevalence of HIV varies depending on rural or urban location, although such variation among in-school youths may be off-set by the homogeneous nature of government schools and the proximity of urban and rural areas in specific regions. The sample population of the current study represented individuals of rural and urban residential status almost equally. Our findings provide evidence that the level of engagement in high risk sexual practices, specifically premarital sex and low condom use, remains considerable among Swazi in-school youths. Such findings are comparable with previous findings from nationwide behavioral surveys in Swaziland, indicating that a substantial number of in-school youths are sexually active but rarely use condoms, particularly at first sexual intercourse, despite their high level of knowledge on HIV prevention [40], [41]. This is a serious concern, as young people are more vulnerable to STIs and HIV (particularly on the first sexual encounter) due to possible abrasion and tearing of the immature reproductive tract. High risk sexual behaviors in Swaziland may be condoned by deep-rooted socio-cultural practices. For instance, Swazi males are often encouraged to exercise their rights to multiple sexual partners, which signifies their power and masculinity, with females being conditioned to submit to men’s leadership and to accept men’s infidelity and polygamy [42], [43]. HIV youth programs in Swaziland continually strive to reduce participation in high risk sexual behaviors among young people. However, the widely-used ABC (Abstain, Be faithful and Condomize) framework largely focuses on individual behavior while neglecting traditional and socio-cultural influences on behavior change.

### Theory of Planned Behavior

Given that, to date, there is limited empirical data supporting the applicability of theory based models in Swaziland, this study provides proof of the applicability of the TPB model among the in-school youth. The TPB model used in this study aims at striking the attention of other researchers who may be motivated to replicate this study to other populations such as out-of school youth and college students in different settings or in different culture. The study advances theoretical research by presenting a different approach of modeling the TPB. This is the first study to utilize the TPB to investigate factors underlying intentions for condom use and abstinence among the Swazi in-school youth. Based on modification indices and theoretical considerations, the final model dichotomized the study’s endogenous variable – protective sexual behavior intention – to two specific intentions: condom use and abstinence. Such an approach is relevant in Swaziland, a country with deeply-rooted cultural elements where young people rarely use condoms regardless of their preference to abstain. Such behaviors stem from various structural factors that can have great influence on sexual behavior in young people.

Preparing adolescents (both virgins and non-virgins) to avoid negative consequences, e.g. by providing information on condom use, trains them to adopt and maintain safer behavioral practices in their future sexual relations regardless of opposing social pressures. The current study simultaneously investigated young people’s intentions to abstain and to use condoms in their future sexual relations. Efforts directed toward reducing the spread of HIV among young people therefore warrant a scaling-up in order to promote both sexual abstinence and condom use, so that in-school youths who ultimately do not abstain are more likely to use condoms in their sexual relations. Results from this study suggest that the TPB may be effective in guiding sex education youth programs based on the study’s good model fit and significant hypothesized paths.

### Predictors of Intentions to Abstain from Premarital Sex

In general, our findings demonstrate TPB variables as important predictors of intentions to abstain from premarital sex; in particular, subjective norms appear to be the strongest predictors of sexual abstinence (effect coefficient = 0.41). This finding is consistent with previous TPB studies, which have demonstrated social approval to be among the most important predictors of adolescent sexual abstinence [4], [7], [44]. Among all subjective norms, parental norms were the most influential toward young people’s intentions to abstain, highlighting the level of influence that parents can have in enhancing effective promotion of sexual abstinence among Swazi in-school youths. The positive impact that parents can have on adolescent sexual behavior has been emphasized by numerous studies [19], [20], [45] and is also consistent with national survey data in Swaziland, which indicate that parenting and family support are key factors in limiting the spread of HIV [46]. Contextual explanations for these findings could be related to the Swazi traditional norm in girls of retaining premarital virginity, which promotes sexual abstinence. The possible interpretation is that parents (especially mothers) are expected to instill traditional values and indeed, participation of virgin girls in an annual reed dance is encouraged and attracts maidens from all parts of the kingdom. The failure of girls to attend may even be taken among community members as an indication of loss of virginity, bringing disrepute to the family. In figure 3, findings further revealed a significantly positive association between culture and young people’s intentions to abstain. In Swaziland, as parents and community elders typically bear responsibility for nurturing young people and overseeing their social wellbeing according to Swazi customs and traditions. Swaziland may be one of the few ethnically homogenous countries in Africa which continuously seeks to embrace its culture and traditions.

Attitudes were fairly reliable predictors of intentions to abstain while the least reliable predictors were perceived behavioral controls. The function of “attitude” as an important predictor of the intention to engage in protective sexual behavior was established in previous studies [47], [48], while perceived behavioral control was previously found to be the least effective toward abstinence [7], [22], [49]. The lack of effect of perceived controls on abstinence intentions may be related to the fact that most in-school youths are fully dependent on their parents or guardians, living under strict rules and regulations without being able to exercise their own intentions. Due to lack of transparency and open communication with their parents, young people are often denied opportunities to exercise voluntary control, instead being required to carry out instructions for fear of punishment. Yet rather than just being prescribed, protective sexual behaviors should be instilled and internalized by young people in order to enable them to independently develop the capacity to refrain from risky sexual practices in the absence of guidance from parents/guardians.

### Predictors of Intentions to Use Condoms

All theory constructs predicted the intention for condom use as hypothesized. Norms of condom use were among the most significant predictors of intention to use condoms (effect coefficient = 0.37). Among all subscales, peer norms were more influential toward the utilization of condoms, followed closely by mother norms, while father norms were least influential. The high influence of peer pressure on adolescent sexual behavior is supported by several studies [50], [51]. Moreover, fathers have previously been found to be the least effective in enforcing intentions toward condom use among their children [47]. The important influence of peers on condom use intentions was expected since, in Swaziland, it remains taboo for young people to talk about contraceptive use in the presence of parents and elders, who expect them to keep their virginity until marriage. Indeed, parents may feel that it is inappropriate to talk to their children about contraception due to socio-cultural norms, and parent-child sex communication remains controversial in Swaziland. Consequently, young people are often limited to discussions on this subject with friends. Furthermore, the lower weighted influence of father norms on condom use intentions may be linked to their passive parental role, as mothers assume a more active role in guiding and ensuring the child’s wellbeing. Fathers may in turn find themselves less apt and not as confident in communicating with their children on sexual and reproductive health topics. These findings highlight the need for parental programs designed to empower parents – especially fathers – on how to effectively monitor and communicate with their children on sensitive issues such as condom use.

Perceived condom use controls were also found to be significant predictors of condom use intentions. Indeed, availability, acceptability, and accessibility of condoms to adolescents have significantly promoted condom utilization in various contexts [52]. In Swaziland, young people still face difficulty in accessing contraceptive services from healthcare facilities for fear of being harshly judged by healthcare personnel upon discovery of their sexual activity. This accentuates the presence of stereotypic healthcare professionals who are still unable to accept the reality of sexual activity among young people, despite the fact that teenage pregnancy, school drop-outs, STIs, and HIV infection are on the rise in this population. Nevertheless, evidence suggests that successful promotion of safer sex among Swazi in-school youths lies in improving condom acceptability and accessibility by overcoming controversial and stereotypic perceptions regarding condom use among young people.

Studies revealed a higher predictive weight for attitude as a predictor of safer sex [22], [47]. In this case, the weak influence for attitudes on condom use intentions may be related to circulation of myths regarding condom use among Swazi youths, such as (1) assumptions that condoms have been injected with the HIV virus and are perpetuating the epidemic, and that (2) use of male condoms results in reduced fertility, as well as being associated with (3) diminished sexual pleasure. Consequently, most Swazi men prefer unprotected sex. These studies show that efforts to positively address attitudes toward condom use among Swazi young people, aimed in particular at confronting misconceptions about condoms, have the potential to play a valuable role in promoting safer sex.

This study has several limitations; firstly, the causal direction between intention and actual behavioral actions of participants requires alternate methodologies such as a longitudinal study design and could therefore not be examined in this cross-sectional study. Secondly, the sensitive nature of the topic may have led to an under-reporting of sexual behavior; although given that the questionnaire was anonymous and completed independently in a classroom under exam conditions, without consulting or discussing questions with classmates, the likelihood of study participants recording inaccurate information was minimal. Thirdly, current findings may not be applicable to other groups of young people such as out-of-school youths, or college and university students. However, the high response rate and robust sampling approach (stratified random sampling) enhance the relevance of the findings to a wider range of the general population. Finally, considering that the sample consisted of virgins and non-virgins, there may have been possible bias emanating from past sexual experience among non-virgins. Another study, however, did not reveal a difference in intentions for condom use between sexually experienced and sexually inexperienced Dutch adolescents [15]. This study contributes overall valuable findings that support the applicability of the TBP within an African context, as in Swaziland. Future longitudinal studies are necessary to verify the causal path between intention and behavior, since behavioral change interventions based on the TPB model can only be effective if the intentions are directly translated to behavior. Intervention studies are also required to determine the effectiveness of HIV prevention programs that address the sexual behavior of young people based on the TPB. The finding that parental norms have significant positive effects on intentions to abstain in young people calls for a deeper understanding of how this comes about. Gender-specific studies are necessary to inform sex education programs in Swaziland and thus, the model needs to be tested in separate female and male subgroups of in-school youths.

### Conclusions

Our findings support the applicability of the TPB in predicting factors that determine sexual behavior in youths in Swaziland, as shown by adequate model fit and significant hypothesized paths. Furthermore, our findings reveal that subjective norms have a vital role in promoting both intention for condom use and abstinence and highlight the effect of parents in positively influencing adolescent sexual behavior.

## References

[1] UNAIDS (2009) Swaziland country report on monitoring the political declaration on HIV and AIDS. Swaziland: Ministry of Health.

[2] World AIDS Day Report (2011) How to get to zero: Faster. Smarter. Better. The UNAIDS vision (Joint United Nations Programme on HIV/AIDS UNAIDS). Geneva, Switzerland.

[3] Makiwane M, Makomane Z (2012) South Africa youths' higher risk sexual behavior: An eco-developmental analysis. African Journal of AIDS Research 9: 17–24. 10.2989/16085906.2010.484538 25860409

[4] Patrick ME, Palen LA, Caldwell L, Gleeson S, Smith E, et al (2010) A qualitative assessment of South African adolescents' motivations for and against substance use and sexual behavior. Journal of Research on Adolescence 20: 456–481. 21625403 10.1111/j.1532-7795.2010.00649.x PMC3101481

[5] Wand H, Ramjee G (2012) The relationship between age of coital debut and HIV seroprevalence among women in Durban, South Africa: A cohort study. BMJ Open 2: e000285. 10.1136/bmjopen-2011-000285 PMC3253418 22223838

[6] Carmack CC, Lewis-Moss RK (2009) Examining the theory of planned behavior applied to condom use: the effect-indicator vs. causal-indicator models. Journal of Primary Prevention 30: 659–676. 19949867 10.1007/s10935-009-0199-3 PMC2872505

[7] Potard C, Courtois R, Samedy ML, Mestre B, Barakat MJ, et al (2012) Determinants of the intention to use condoms in a sample of French adolescents. European Journal of Contraception and Reproductive Health Care 17: 55–64. 22149900 10.3109/13625187.2011.634455

[8] Farmer MA, Meston CM (2006) Predictors of condom use self-efficacy in an ethnically diverse university sample. Archives of Sexual Behavior 35: 313–326. 16804746 10.1007/s10508-006-9027-5 PMC2859307

[9] Fatusi AO, Blum RW (2008) Predictors of early sexual initiation among a nationally representative sample of Nigerian adolescents. BMC Public Health 8: 136. 18439236 10.1186/1471-2458-8-136 PMC2390536

[10] Sayles JN, Pettifor A, Wong MD, MacPhail C, Lee S, et al (2006) Factors associated with self-efficacy for condom use and sexual negotiation among South African youth. Journal of Acquired Immune Deficiency Syndrome 43: 226–233. 10.1097/01.qai.0000230527.17459.5c PMC2819666 16951647

[11] Bailey JA, Fleming CB, Henson JN, Catalano RF, Haggerty KP (2008) Sexual risk behavior 6 months post-high school: Associations with college attendance, living with a parent, and prior risk behavior. Journal of Adolescent Health 42: 573–579. 18486866 10.1016/j.jadohealth.2007.11.138 PMC5812449

[12] Baumgartner SE, Valkenburg PM, Peter J (2010) Assessing causality in the relationship between adolescents' risky sexual online behavior and their perceptions of this behavior. Journal of Youth and Adolescence 39: 1226–1239. 20177962 10.1007/s10964-010-9512-y PMC2917006

[13] Henderson M, Butcher I, Wight D, Williamson L, Raab G (2008) What explains between-school differences in rates of sexual experience? BMC Public Health 8: 53. 18261205 10.1186/1471-2458-8-53 PMC2277387

[14] Van Rossem R, Meekers D (2011) Perceived social approval and condom use with casual partners among youth in urban Cameroon. BMC Public Health 11: 632. 21819611 10.1186/1471-2458-11-632 PMC3170619

[15] Swaziland National Network of People Living with HIV and AIDS (2011) Best practices on challenging gender dynamics in cultural contexts: SWANNEPHA and NATICC implementing the ‘changing the river’s flow’ programme’ in Swaziland (NATICC & SWANNEPHA Report).

[16] Uwah C, Wright S (2012) Socio-cultural identities, perceptions of sexuality/sexual behavior and cultural contexts as determinants of HIV and AIDS prevalence in Southern Africa. World Journal of AIDS 2: 17–23.

[17] Villarruel AM, Jemmott JB 3rd, Jemmott LS, Ronis DL (2004) Predictors of sexual intercourse and condom use intentions among Spanish-dominant latino youth: A test of the planned behavior theory. Nursing Research 53: 172–181. 15167505 10.1097/00006199-200405000-00004

[18] Brauner-Otto SR, Axinn WG (2010) Parental family experiences, the timing of first sex, and contraception. Social Science Research 39: 875–893. 21079724 10.1016/j.ssresearch.2010.06.015 PMC2978908

[19] De La Rosa M, Dillon FR, Rojas P, Schwartz SJ, Duan R (2010) Latina mother-daughter dyads: Relations between attachment and sexual behavior under the influence of alcohol or drugs. Archives of Sexual Behavior 39: 1305–1319. 19399605 10.1007/s10508-009-9498-2 PMC2891179

[20] Gardner M, Martin A, Brooks-Gunn J (2012) Exploring the link between caregiver affect and adolescent sexual behavior: Does neighborhood disadvantage matter? Journal of Research on Adolescence 22: 135–149. 22408364 10.1111/j.1532-7795.2011.00752.x PMC3293489

[21] Busse P, Fishbein M, Bleakley A, Hennessy M (2010) The role of communication with friends in sexual initiation. Communication Research 37: 239–255. 20613973 10.1177/0093650209356393 PMC2897170

[22] Selikow TA, Ahmed N, Flisher AJ, Mathews C, Mukoma W (2009) I am not "umqwayito'': A qualitative study of peer pressure and sexual risk behaviour among young adolescents in Cape Town, South Africa. Scandinavian Journal of Public Health 37 Suppl 2107–112. 19493988 10.1177/1403494809103903

[23] Bleakley A, Hennessy M, Fishbein M, Jordan A (2009) How sources of sexual information relate to adolescents’ beliefs about sex. American Journal of Health Behavavior 33: 37–48. 10.5993/ajhb.33.1.4 PMC2860278 18844519

[24] Monahan KC, Steinberg L, Cauffman E (2009) Affiliation with antisocial peers, susceptibility to peer influence, and antisocial behavior during the transition to adulthood. Developmental Psychology 45: 1520–1530. 19899911 10.1037/a0017417 PMC2886974

[25] Stevensons J (1996) Applied multivariate statistics for the social sciences. Lawrence Eelbaum: Mahwah, NJ.

[26] Swazi Government Report (2011) School Lists. Mbabane, Swaziland: Ministry of Education.

[27] Joint United Programme on HIV/AIDS (2007) Swaziland HIV/AIDS and projections. Mbabane, Swaziland: NERCHA, UNAIDS, Ministry of Health and Social Welfare.

[28] National Emergency Council on HIV and AIDS (2009) Swaziland HIV prevention response and modes of transmission analysis. Mbabane, Swaziland: Swazi Government.

[29] Philippa C (1996) Age differences in parent and peer influences on female sexual behavior. Youth Studies Australia 3: 60.

[30] Cha ES, Doswell WM, Kim KH, Charron-Prochownik D, Patrick TE (2007) Evaluating the Theory of Planned Behavior to explain intention to engage in premarital sex amongst Korean college students: A questionnaire survey. International Journal of Nursing Studies 44: 1147–1157. 16814789 10.1016/j.ijnurstu.2006.04.015

[31] Cha ES, Kim KH, Patrick TE (2008) Predictors of intention to practice safer sex among Korean college students. Archives of Sexual Behavior 37: 641–651. 17680355 10.1007/s10508-007-9187-y

[32] Basen-Engquist K, Masse LC, Coyle K, Kirby D, Parcel GS, et al (1999) Validity of scales measuring the psychosocial determinants of HIV/STD-related risk behavior in adolescents. Health Education Research 14: 25–38. 10537945 10.1093/her/14.1.25

[33] Norris AE, Clark LF, Magnus S (2003) Sexual abstinence and the sexual abstinence behavior scale. Journal of Pediatric Health Care 17: 140–144. 12734461 10.1067/mph.2003.12

[34] Hanna KM (1999) An adolescent and young adult condom self-efficacy scale. Journal of Pediatric Nursing 14: 59–66. 10063250 10.1016/S0882-5963(99)80061-X

[35] Doswell WM, Kim Y, Braxter B, Taylor J, Kitutu J, et al (2002) A theoretical model of early teen sexual behavior: What research tells us about mother's influence on the sexual behavior of early adolescent girls. Journal of Theory Construction & Testing 7: 56–60.

[36] Schumacher RE, Lomax RG (2010) A beginner’s guide to structural equation modeling (3rd ed.). Mahwah, NJ: Lawrence Erlbaum Associates.

[37] MacCallum RC, Widaman KF, Zhang S, Hong S (1999) Sample size in factor analysis. Psychological Methods 4: 84–99.

[38] Kline RB (1998) Principles and practice of structural equation modelling. New York: NY: The Guilford Press.

[39] Swaziland Behavioral Surveillance Survey (2002) Swaziland Behavioral Surveillance Survey. Swaziland Ministry of Health and Social Welfare.

[40] Swaziland Second Behavioral Surveillance Survey (2010) Report on the Second Behavior Surveillance Survey Adult and Youth Component Progress (Swaziland Ministry of Health Report). Swaziland Ministry of Health and Social Welfare.

[41] Mathunjwa TR, Garey FA (2006) Women and HIV/AIDS in the kingdom of Swaziland: culture and risks. Journal of National Black Nurses Association 17: 39–46. 17410758

[42] Tobias BQ (2001) A descriptive study of the cultural mores and beliefs toward HIV/AIDS in Swaziland, Southern Africa. International Journal for the Advancement of Counselling 23: 99–113.

[43] Myklestad I, Rise J (2007) Predicting willingness to engage in unsafe sex and intention to perform sexual protective behaviors among adolescents. Health Education & Behavior 34: 686–699. 16885507 10.1177/1090198106289571

[44] Hutchinson MK, Kahwa E, Waldron N, Hepburn Brown C, Hamilton PI, et al (2012) Jamaican mothers' influences of adolescent girls' sexual beliefs and behaviors. Journal of Nursing Scholarship 44: 27–35. 22339731 10.1111/j.1547-5069.2011.01431.x PMC3288879

[45] UNAIDS (2012) Swaziland Country Report on Monitoring the Political Declaration on HIV and AIDS. Swaziland: Ministry of Health.

[46] Alvarez C, Villarruel AM, Zhou Y, Gallegos E (2010) Predictors of condom use among Mexican adolescents. Research and Theory for Nursing Practice 24: 187–196. 20949835 10.1891/1541-6577.24.3.187 PMC3290769

[47] Doswell WM, Braxter BJ, Cha E, Kim KH (2011) Testing the theory of reasoned action in explaining sexual behavior among African American young teen girls. Journal of Pediatric Nursing 26: e45–54. 22055383 10.1016/j.pedn.2011.03.007

[48] Molla M, Astrøm AN, Berhane Y (2007) Applicability of the theory of planned behavior to intended and self-reported condom use in a rural Ethiopian population. AIDS Care 19: 425–431. 17453579 10.1080/09540120600722692

[49] Warner TD, Giordano PC, Manning WD, Longmore MA (2011) Everybody’s doin’ it (right?): Neighborhood norms and sexual activity in adolescence. Social Science Research 40: 1676–1690. 22427712 10.1016/j.ssresearch.2011.06.009 PMC3302666

[50] Zwane IT, Mngadi PT, Nxumalo MP (2004) Adolescents’ views on decision-making regarding risky sexual behaviour. International Nursing Review 51: 15–22. 14764010 10.1111/j.1466-7657.2003.00214.x

[51] Hendriksen ES, Pettifor A, Lee SJ, Coates TJ, Rees HV (2007) Predictors of condom use among young adults in South Africa: The reproductive health and HIV research unit national youth survey. American Journal of Public Heaith 97: 1241–1248. 10.2105/AJPH.2006.086009 PMC1913066 17538062

[52] Lee YH, Salman A, Fitzpatrick JJ (2009) HIV/AIDS preventive self-efficacy, depressive symptoms, and risky sexual behavior in adolescents: A cross-sectional questionnaire survey. International Journal of Nursing Studies 46: 653–660. 19159880 10.1016/j.ijnurstu.2008.11.007

